# Barriers and facilitating factors related to use of early warning score among acute care nurses: a qualitative study

**DOI:** 10.1186/s12873-017-0147-0

**Published:** 2017-12-01

**Authors:** John Asger Petersen, Lars S. Rasmussen, Susan Rydahl-Hansen

**Affiliations:** 1 Department of Day Surgery, Hvidovre University Hospital, Kettegaards Alle 30, 2650 Hvidovre, Denmark; 2 Center of Head and Orthopedics, Rigshospitalet, University of Copenhagen, Blegdamsvej 9, 2100 Copenhagen Ø, Denmark; 3 Department of Public Health - Department of Science in Nursing, Aarhus University, Campus Emdrup Tuborgvej 164, 2400 Copenhagen, NV Denmark

## Abstract

**Background:**

The early warning score (EWS) was developed to identify deteriorating patients early. It is a track-and-trigger system based on vital signs designed to direct appropriate clinical responses based on the seriousness and nature of the underlying condition. Despite its wide dissemination, serious adverse events still occur, often due to failure among staff on general wards to follow the EWS protocol. The purpose of the study was to determine barriers and facilitating factors related to three aspects of the EWS protocol: 1) adherence to monitoring frequency, 2) call for junior doctors to patients with an elevated EWS, and 3) call for the medical emergency team.

**Methods:**

Focus groups were conducted with nurses from medical and surgical acute care wards, and content analysis was used to identify barriers and facilitating factors in relation to the research questions.

**Results:**

Adherence to monitoring frequency would frequently be set aside during busy periods for other tasks. Collaboration and communication with doctors about medical patients with elevated EWS was considered to be unrealistic due to the high number of patients with these scores. Collaboration with the medical emergency team was problematic, since many nurses found the team to have negative attitudes.

**Conclusion:**

EWS reduces complex clinical conditions to a single number, with the inherent risk to overlook clinical cues and subtle changes in patients’ condition. The study showed that identifying and treating deteriorating patients is a collaborative task that requires diverse technical and non-technical skills for staff to perform optimally.

## Background

The use of Rapid Response Systems (RRS) is widespread in hospitals in UK, USA, Australia, and Scandinavia. RSS are tools to identify patients at risk of clinical deterioration and intervene appropriately to prevent serious adverse events (SAE), such as cardiac arrest, unexpected death, and unanticipated ICU admission [1]. Staff on general wards act as the afferent limb of the RRS, to detect at-risk patients and alert members of the efferent limb, usually a medical emergency team (MET) manned with specialists in emergency medicine or critical care, to treat the patient [2]. The early warning score (EWS) was developed as an aid for general ward staff to detect deterioration [3]. It is based on a set of routinely measured vital signs (Table 1) and clinical triggers to direct staff as to when and how to escalate care (Table 2). To ensure efficiency and appropriateness of the response, the escalation process is condensed to a treatment protocol that prescribes the actions and competency of the providers according to the severity of deviations of the vital signs from a predefined normal range [3, 4]. To be successful, every level of the EWS protocol has to be properly executed. However, this is not always the case. An analysis of SAE at our institution showed full compliance with the escalation protocol in only 12 (8%) of 144 cases. Specifically, monitoring was delayed in 81% of the cases, and in patients with EWS ≥ 3 and EWS ≥ 6, nurses and physicians escalated care appropriately in only 60% and 41% respectively. Furthermore, only 50% of patients with EWS ≥ 9 were reviewed by MET or senior staff [5]. Correspondingly, other studies have identified afferent limb failure as a major problem in 20–80% of serious adverse events [6–8]. Thus, it is well established that afferent limb failure does occur, but the reasons behind it are less clearly investigated. A study from the USA found that only 25% of staff adhered to calling criteria for MET and resistance to do so correlates with lack of knowledge and negative attitudes towards MET [9]. Conversely, an Australian study showed non-adherence in 42% of cases, but here the main barrier was the staff’s confidence in their own abilities to handle patients without assistance [10]. Other studies have identified a range of other barriers, including lack of knowledge and negative views on RRS, fear of criticism from the MET, or fear to appear incompetent [11, 12]. Most of the existing studies examine nurses’ barriers to MET call, and, to our knowledge, no studies have investigated barriers to adherence to the other steps in the EWS protocol. Nurses are the first line responders in the stepwise process from identification to treatment of deteriorating patients. Their ability to identify at-risk patients and respond appropriately is pivotal for the subsequent steps of the escalation protocol to be executed successfully. Therefore, the aim of the present study was to identify barriers and facilitating factors related to use of the EWS escalation protocol among nurses.

**Table 1:** Early warning score with physiological parameters and corresponding weighted score and normal range used in theCapital Region of Denmark

Vital sign	3	2	1	0	1	2	3
Respiratory Rate pr min	<9		9–11	12–20		21–24	>24
Oxygen saturation	<92%	92–93%	94–95%	>95%			
Supplemental oxygen		YES		No			
Temperature degrees centigrade	<35.1		35.1–36.0	36.1–38.0	38.1–39.0	>39	
Systolic blood pressure mmHg	<91	91–100	101–110	111–219			>219
Heart rate pr min	<41		41–50	51–90	91–110	111–130	>130
Level of consciousness				A			V, P, U

AVPU = alert, verbal response, responsive to pain, unresponsive

**Table 2:** EWS thresholds and clinical responses to triggers used in the Capital Region of Denmark

EWS	Frequency of monitoring	Clinical response according to escalation protocol
0–1	Minimum 12 hourly	• Continue monitoring minimum 12 hourly
2	Minimum 6 hourly	• Assessment of airway, breathing and circulation and appropriate intervention
3–5	Minimum 4 hourly	• Assessment of airway, breathing and circulation and appropriate intervention • Nurse in charge informs on-call physician, who assesses patient and plans appropriate treatment and/ or diagnostics
6	Minimum 4 hourly	• Assessments of airway, breathing and circulation and intervenes appropriately • Urgent assessment by on-call physician, including plan for appropriate treatment and diagnostics
7–8	Minimum 1 hourly	• Assessment of airway, breathing and circulation and appropriate intervention • Emergency assessment (within 30 min) by on-call physician, including plan for appropriate treatment and diagnostics • Consider call to medical emergency team (MET)
≥ 9	Minimum ½ hourly	• Assessment of airway, breathing and circulation and appropriate intervention • Emergency assessment (within 15 min) by on-call physician, including plan for appropriate treatment and diagnostics • Patient must be evaluated with senior physician or MET

## Methods

### EWS system and MET

EWS is recommended for use in the UK and a modified version has been in use at hospitals in the Capital Region of Denmark since 2013 [4, 5]. Each vital sign can be assigned between 0 and 3 points (supplementary oxygen 0 or 2) and values are added to an aggregated score from 0 to 20, higher scores indicating more severe disease (Table 1). A mandatory escalation protocol directs type of clinical response and responsibility of the provider and is an integrated part of the system (Table 2). Monitoring frequency increases with higher scores. Unlike UK guidelines, where urgent or immediate review by a physician or MET is mandated at scores of 5 and 7 respectively, at our institution scores 3–5 mandate nurses to inform the on-call physician, who must assess the patient and document additional treatment and diagnostic plans. Patients with scores of 6–8 must be evaluated by a physician immediately, and EWS ≥ 9 mandates evaluation by a senior physician or MET without delay.

EWS was implemented at our institution in 2012 through involvement of specially trained members of the nursing staff and physicians together with heads of departments. Presently all new employees are introduced to the system and there is ongoing training for all healthcare providers on general wards in assessment and initial stabilization of acutely deteriorating patients.

MET has been implemented since 2007. It is manned with a specially trained ICU nurse and a senior registrar or staff specialist in anesthesia. Request for MET review is done by telephone directly to the ICU nurse. All patients are eligible for MET call, regardless of EWS, and all healthcare staff is allowed to call MET, if they are concerned.

### Study design

Focus groups were conducted with nurses from the medical and surgical acute care wards at Bispebjerg-Frederiksberg University Hospital to identify barriers and facilitating factors related to the following aspects of the EWS escalation protocol:

1) What are the barriers and facilitating factors in relation to adhering to monitoring frequency?

2) What are the barriers and facilitating factors in relation to informing doctors at EWS ≥ 3?

3) What are the barriers and facilitating factors in relation to initiating MET calls?

Because they reflect crucial nursing tasks of the EWS protocol to identify at-risk patients and escalate care appropriately.

### Setting

Our institution is an urban 700 beds hospital serving a population of approximately 400,000 in the Capital Region of Copenhagen, Denmark. The surgical acute care ward has 20 beds and receives approximately 6500 acutely admitted abdominal surgery patients annually, and is staffed by 26 nurses. The medical acute care ward has 36 beds, and an annual intake of approximately 7500 patients and 55 nurses. Both wards receive patients referred from general practitioners, the emergency department, out-patient clinics, or other wards.

### Participants

To ensure familiarity with the EWS protocol, it was decided before start of the study that only nurses with at least three months of employment on the ward were eligible to participate. Potential participants were nominated by the head nurses of each ward, informed about the study, and asked if they would like to participate voluntarily. Focus groups were conducted during working hours in a quiet meeting room separate from the wards, and scheduled to take approximately 90 min with a total of 3–6 participants. No stratification was conducted, instead we aimed to include two nurses from each ward in a focus group. The main author (JAP), an ICU physician with experience in emergency medicine, was the main moderator and all focus groups were also supervised by an external nurse researcher with extensive experience in qualitative interview technique. The aim of the focus groups was to facilitate an open discussion among group members about the three research questions [13]. An interview guide was prepared beforehand in an iterative process by the authors to ensure that all aspects of use of the escalation protocol were addressed (Table 3) [14]. It consisted of general questions about ward routines in regard to care and diagnosis of deteriorating patients to engage participants in the discussion, and subsequently focused specifically on barriers and facilitating factors related to patient monitoring, cooperation with junior doctors and the MET (Table 3). Participants finally were asked for suggestions to overcome the obstacles identified during the interviews. Only short digressions from the topic were allowed and both moderators were attentive to keep discussion on track and cover relevant topics. Focus groups were conducted in Danish and quotes translated by the main author (JAP). All interviews were recorded and transcribed verbatim.

**Table 3:** Interview guide for focus group interviews

Themes	Interview questions
Briefing and introduction	• Introduction of the interviewers and aim of the interview • Briefing that participation in the interview is voluntary and data will be published anonymized • I ask the participants to briefly introduce themselves by name, place of employment, and how long they have been nurses and worked on the ward
General aspects of knowledge and understandings of acutely deteriorating patients	• In your opinion, what is an acutely deteriorating patient? ○ Try to describe your last acutely deteriorating patient. ○ What connections do you see between critical illness and patients’ diagnosis? ○ What connections do you see between critical illness and patients’ vital signs? ○ What connections do you see between critical illness and progression in patients’ condition? ○ What connections do you see between critical illness and patients’ co-morbidities? ○ In your opinion, are there any other findings that lead you to conclude that a patient is acutely deteriorating?
General aspects of handling acutely deteriorating patients on the wards	• Try to tell how you typically handle acutely deteriorating patients on your ward ○ What is the role of other nurses? ○ How do you delegate tasks between doctors and nurses on your wards? ○ How do you typically identify at risk patients on your wards? ○ How do you determine how close patients should be monitored on your wards? ○ How do you decide what interventions and treatments acutely deteriorating patients receive on your wards? ○ How do you determine if you need further assistance to handle acutely deteriorating patients on your wards?
General aspects of the role of early warning score in identifying and handling acutely deteriorating patients	• In your opinion, what is the role of EWS and the related algorithm in handling acutely deteriorating patients? ○ Try to describe if and when you use EWS in identifying acutely deteriorating patients. ○ Try to describe if and when you use EWS in monitoring acutely deteriorating patients. ○ Try to describe if and when you use EWS in stabilizing acutely deteriorating patients. ○ Try to describe if and when you use EWS to obtain necessary assistance in handling acutely deteriorating patients.
Specifically about barriers and facilitators in relation to adherence to monitoring frequency	• Try to describe what issues make it easy or hard to adhere to the prescribed monitoring frequency of the EWS algorithm. ○ In what circumstances would you typically deviate from the algorithm and monitor more or less frequently? ○ What issues in your daily work life impact on the adherence to the algorithm? • Do you consider it important to adhere to the prescribed monitoring frequency? • What could be done to make it easier to adhere to the prescribed monitoring frequency?
Specifically about barriers and facilitators in relation to inform junior doctors about patients with moderately elevated EWS (≥ 3)	• According to the algorithm junior doctors must be informed about every patient with a moderately elevated EWS of 2–3, what do you think of that? ○ How often do you inform junior doctors about these patients? ○ Under what circumstances do you inform junior doctors about these patients? ○ When do you decide not to inform them? ○ What issues in your daily work life impact on the adherence to the algorithm? • In your opinion, what is the most important issue that determines whether you do or do not inform junior doctors? • What could be done to make it easier to inform junior doctors?
Specifically about barriers and facilitators in relation to MET calls	• Try to describe when you last made a MET call? • In what circumstances do you use MET? ○ Are there specific categories of patients where you call MET? ○ Are there specific times of the day when you use MET? ○ What criteria do you use to determine whether to call for MET or not? ○ What issues in your daily work life impact on the adherence to the algorithm in regard to MET calls? • When do you not use MET? • What is the role of EWS in your decision to call MET? • In your opinion, what is the most important issue that determines whether you do or do not inform junior doctors? • What could be done to make it easier to use MET?
Debriefing	• Are there any important issues we need to talk about in regard to acutely deteriorating patients? • Thank you for your participation.

### Analysis and validity

Analysis was based on Krippendorff’s components of text driven content analysis: unitizing, sampling, coding, reducing, and abductively and inductively inferring contextual phenomena, and finally formulating answers to the research questions, i.e. find explanations that are simple, coherent, and in line with prior knowledge. [15].

Analysis was performed on data from interview transcripts in the following way: first through immersion in the text by reading it several times, to get a sense of the participants perspectives; secondly text was divided into meaningful units in relation to the original research questions, concerning monitoring frequency, alerting junior doctors, and MET calls and coded by one of the authors (JAP) in collaboration with one of the other author (SRH). Finally codes with corresponding contents were merged into subcategories, and formulated into meaningful main categories in relation to the research questions, e.g. statements and remarks about difficulties to comply with monitoring intervals or reluctance to engage MET in treatment decisions would be categorized as barrier to comply with monitoring frequency and barrier to call MET, respectively [15, 16].Validity of our findings was sought throughout the research process through methodological coherence, appropriate sampling, collecting and analyzing data to answer the research questions [17]. Briefly, this was ensured by conducting interviews until data saturation was achieved, and the two facilitators conducting the interviews judged that no new information emerged. To ensure data completeness the main moderator (JAP) facilitated the discussion while the secondary moderator concurrently checked the interview guide (Table 3) and supplemented with further questions to cover all research questions sufficiently. RDQA, an R package for Qualitative Data Analysis, was used to perform coding and categorization of the transcripts.

## Results

We enrolled 18 nurses (2 male, 16 female); 7 surgical (26% of total nurse staff) and 11 medical (20% of total nurse staff) with tenure from 1.5 to 22 (median 2.8, interquartile range 1.6 to 4.0) years. A total of five focus group interviews were conducted from July 20 to October 29, 2014 with 3 to 5 participants in each. No nurses refused to participate, but one focus group was conducted with only medical staff, due to a busy surgical ward.

In the following, the main categories that emerged during the content analysis are presented in relation to the original three research questions.

### Research question 1: What are the barriers and facilitating factors in relation to adhering to monitoring frequency?

Two forms of non-adherence to monitoring frequency emerged during the interviews: over-monitoring, i.e. monitoring more frequently than per protocol, was primarily described in positive terms, while under-monitoring generally was viewed as objectionable and considered bad nursing practice, but reported to occur frequently during busy periods.

#### Over-monitoring out of concern for the patient

The decision to monitor more often than required by protocol was usually based on a gut feeling, sixth sense, or clinical intuition. However, on further scrutiny the feeling was usually grounded in clinical and diagnostic cues not included in EWS e.g., skin color, respiratory pattern, diaphoresis, patient’s self-reported feeling of distress, and others. Other commonly cited triggers in absence of elevated EWS were clinical conditions with high risk of deterioration, including severe bleeding, intoxication or early stage of sepsis. Changes in vital signs within the threshold limits, e.g. changes in respiratory frequency from 25 to 44 that would not increase the overall EWS, could also prompt nurses to increase monitoring.

“(…)we [nurses] use our clinical intuition to see the patient an extra time and take an extra set of vitals, because you have some alarm bells ringing. If something just doesn’t seem right I prefer to take an extra EWS score even though nothing sticks out, because there is something you just can’t define (…)”.

A prominent concern was assessment of level of consciousness with the AVPU (alert – response to verbal stimuli – response to pain – unresponsive) scale in patients with pre-existing mental or psychiatric disorders or delirium, since even severe decrease in level of consciousness would not always be reflected by increase in EWS in these patients.

Generally, over-monitoring was considered a good thing as long as it was based on nurses own feeling of concern and in concordance with their assessment of the situation. However, nurses could question the benefit of monitoring more frequently than per protocol, if it was prescribed by junior doctors. In these instances it was described as interfering with work flow and unnecessary.

“I actually had the (…) debate [*concerning monitoring frequency*] with several of our junior doctors, because they feel a huge responsibility (…) and for no reason they prescribe to measure EWS every two hours in a young totally healthy patient with EWS 0. I try to argue (…) you got to trust me on this; I’ll look after the patient and if it gets worse I’ll take an extra EWS, but otherwise we follow the [*escalation*] protocol.”

#### Lack of resources as a reason for under-monitoring

Monitoring less frequently than prescribed was generally not favored, but occurred regularly during busy periods. Under-staffing and time constraints were reported, as the main barriers for this form of non-adherence to monitoring protocol. Interestingly, these constraints were accepted as a fundamental and unalterable condition of work life, and the nurses had devised a number of strategies to handle these situations. The guiding principle was to treat the sickest patient first. Triage was based on nurses’ clinical assessment of the patient’s condition, with EWS as one among several clinical tools, others being: diagnosis on admission, severity of symptoms, and acuity of the underlying condition. Nurses generally stated that patients with EWS ≥ 7 were considered high-risk, and prioritized over patients with lower EWS, unless special circumstances dictated otherwise. However, nurses from the surgical ward typically considered patients with lower EWS, around four, as high risk. This was primarily attributed to different case-mix between wards, with a higher proportion of elderly, frail patients on the medical ward. Triage was a collaborative process performed in cooperation with other nurses and physicians; there were no guidelines for these situations, but non-formal discussions about general principles for task prioritization occurred on staff meetings. Nurses had adopted approaches to secure patient safety. Typically by covering for each other, so nurses with the sickest patients would not be distracted by routine tasks, this could gradually be escalated to calling the MET for assistance. However, no clear trigger for how and when to escalate resources existed. During busy periods, adherence to monitoring frequency, was not regarded essential, and often set aside, if it interfered with workflow on the ward, as long as the nurses felt comfortable and considered it safe. Generally patients with high EWS were prioritized over patients with lower scores.

#### Concern about sleep deprivation as reason for under-monitoring

Another frequently mentioned reason to omit monitoring was concern for sleep deprivation; specifically concern to cause or aggravate delirium. There was broad consensus that sleep was important and it was generally accepted to omit monitoring, if the patient was asleep and appeared in no distress. Nurses reported they would observe patients at a distance, careful not to awaken or disturb them. Only few nurses questioned their abilities to distinguish normal sleep from unconsciousness in this way.

#### Practical constraints

During acute emergencies nurses reported that documentation of vital signs, but not monitoring, was neglected. In these situations vital signs were taken frequently and recorded manually on paper. However, these data would usually not be registered electronically in the hospital’s patient data management system, as this was considered a purely bureaucratic task. It was stated that monitoring frequency sometimes was adjusted to fit with shift handovers and other ward routines. This was considered unproblematic and in accordance good practice to facilitate work-flow. Also, patients might be unavailable for monitoring, either because they were out of the ward for examinations, had visitors, or were otherwise engaged.

Practical issues like time constraints, patients unavailable for monitoring and time consuming work with documentation were frequently mentioned as barriers to monitoring; as a result many nurses mentioned technological solutions such as automated continuous surveillance of vital signs as a solution. However, not all nurses agreed, as many viewed the process of measuring vital signs as an important opportunity to observe and interact with patients, and as an important part of their clinical assessment and care.

### Research question 2: What are the barriers and facilitating factors in relation to informing doctors at EWS ≥ 3?

#### EWS in range 3–6 is considered the norm

As a general rule nurses did not inform doctors about patients with moderately elevated EWS in the range from 3 to 6. This was considered as unrealistic due to the great number of patients with moderately elevated EWS, and accordingly nurses felt quite comfortable and competent to handle these patients without assistance.

“Moderator: (…) according to protocol you have to inform the on-call physician at an EWS of 3. Is that something you do?

Nurse 1: No.

Nurse 2: Never.

Nurse 3: Only on arrival.”

They saw no need to inform doctors, since it would rarely change the management of the patient. There was broad consensus that this was a reasonable approach that would not jeopardize patient safety, and furthermore facilitate work flow.

“I have never called [*a doctor*] for an EWS of 3. I think the answer would be: take a new one [*EWS*] in an hour and call me again, if it gets worse. Except, if I had something specific I could put my finger on, I could get him to come, but just an EWS of 3 is not enough.”

Typically, nurses on the medical ward would accept an EWS ≥ 7–9 before consulting the on-call physician. Immediate review was rarely requested; as long as the nurses had the feeling they were in control of the situation. Interestingly, experienced nurses reported they had alternative triggers besides EWS to call for assistance, based on their experience. Younger nurses seemed to have adopted their approach, that is, replace EWS protocol with clinical judgment based on experience, even if limited. Generally, surgical ward nurses had a lower threshold for calling, and perceived their role as more limited in regard to initiating treatments. Their goal would be to alert surgeons early, so patients could be expedited to surgery, or, if there was no underlying surgical condition, arrange patients to be moved to the medical ward for further treatment.

#### The role of interpersonal relations

Interpersonal relations between nurses and doctors were cited as pivotal in patient care and could act as both barriers and enhancers in patient care. A main point of concern was to disrupt doctors with unnecessary calls; and it was commented that a recent introduction of mobile phones, instead of pagers, for doctors had facilitated communication, since they now could answer calls more easily and without delay. Apart from the technological issues, nurses stated that they were more inclined to reach out for doctors they knew beforehand, had good relations with, and considered to be skilled. They were reluctant to call junior doctors and rarely regarded their contributions as valuable or helpful.

### Research question 3: What are the barriers and facilitating factors in relation to initiating MET calls?

There was a prevailing culture to call MET only for patients with an urgent need for higher level of care than the wards could provide. Calls would not be done as per protocol, but were instead based on a combination of the patient’s clinical condition, his or her perceived needs, and available resources on the ward.

#### Attitude of MET

The main barrier related to MET calls was a feeling of anxiety towards the team. While it was acknowledged that collaboration with MET generally was good, most nurses had experienced frustrating, intimidating, or distressing encounters with MET.

“I think it [MET] has helped me a couple of times, when I thought I had done all I could, but suddenly the patient [*deteriorates*] (…) and it was nice to be able to call someone with expertise. But I have sometimes felt that they talked down to me, and asked if I had done this and that. I thought by myself, but we do not have this kind of equipment and I don’t know these things. I mean, I am willing to start things up and give a hand, so sometimes I have felt talked down to and very stupid. But at other times the team [*MET*] is nice and forthcoming and said they were glad we called.”

Some felt they had to negotiate to convince the MET nurse to come to see patients, or were frustrated about giving a lot of background information about patients, before MET reviews could be initiated. The result was that nurses could feel belittled, criticized, or reprimanded. MET was seen as aloof and at times disrespectful.. These feelings were prevalent to a degree that many nurses refrained from initiating calls on their own initiative. Typically, MET calls were seen as a last resort, when all other options were exhausted. A call would always be a collaborative decision between doctors and nurses.

“We [nurses] don’t call them [*MET*], or I haven’t called them. I don’t know anyone [*that has*], except if one of the doctors asks us to call. But I have asked doctors, if we shouldn’t call the MET, but they said no. I don’t know, if it is out of fear not to be able to handle the situation themselves or if they are ashamed to ask for help?”

Most nurses valued the know-how and expertise of the MET, and expressed that they would like to have closer collaboration. Not only for reviews, but also for discussion about patients, before they had deteriorated to a degree that demanded transfer to the ICU. However, this was not considered an option due to the negative feelings surrounding the team. They emphasized the importance of non-technical skills to support good teamwork, especially during critical situations, and found this lacking in certain members of the MET.

#### Facilitating factors of MET calls

While non-technical skills of MET was the primary hurdle to MET calls, nurses suggested that training in these skills would enhance collaboration. They suggested joint training sessions and education to strengthen teamwork and relations between ward staff and members of MET and align expectations both ways.

## Discussion

Data from five focus groups with nurses from the surgical and medical acute care wards at our institution revealed a number of barriers and facilitating factors related to the use of the EWS protocol. Generally, EWS and the corresponding escalation protocol were described in positive terms, specifically their usefulness as an aid in clinical assessment, to facilitate inter- and intra-professional communication, and prioritize workload, were emphasized. We identified a number of barriers and facilitators in relation the three research questions.

In regard the first question, it emerged that adherence to protocol was considered an important aspect of professional conduct and part of evidence based practice. It was often performed more frequently than per protocol, primarily out of concern for the patient’s condition based on clinical clues besides EWS. Strictly speaking “over-monitoring” is a deviation of the monitoring protocol, while it is probably not harmful, and most likely beneficial, for the affected patient, it does take time away from other tasks and could inadvertently harm other patients. However, this was not perceived as a problem, if monitoring was initiated out of the nurses’ own concern, but perceived as burdensome when requested by doctors.

Monitoring less frequently than prescribed was generally viewed unfavorably, but occurred regularly during busy periods and at night. Lack of resources was mentioned as one of the main barriers to adequate monitoring, and increased staffing, especially during busy periods, more efficient monitoring, either with automated systems or by limiting monitoring to high risk groups were mentioned as facilitators for better adherence to monitoring frequency. Specifically, nightly monitoring merits further evaluation to assess benefits and drawbacks of sleep disturbance versus protocolled observation.

In regard to research question 2, nurses did not consider following the protocol in regard to informing doctors about patients with EWS ≥ 3 as particularly important. This was mainly due to the number of patients with elevated EWS. An EWS of 3–6 was generally considered low risk, and, during busy periods, these patients would not be observed as strictly as prescribed according to protocol. This is in line with earlier publications where short term mortality within 48 h of admission was found to be at 0.41% in this population of patients. However, since they constitute almost 1/3 of admitted patients, they make up for almost 40% of all deaths during the first two days of admission [18, 19].

In regard to the third research question, we found nurses generally reluctant to call MET and commonly regarded it as a last resort. The main barrier to call MET was the perceived negative attitude of the MET, as many nurses had witnessed inappropriate behavior. This phenomenon is well described in a number of studies, and the importance of continuous education of staff on general wards as well as feedback on performance to the MET and training of non-technical skills is considered an important component of a well-functioning RRS [10, 20–23].

Shortcomings in this study include its single center design and recruitment of nurses from only two wards. This makes it difficult to extrapolate our findings to other institutions. Furthermore, recruitment through head nurses introduces a number of biases and prompts ethical considerations. First and foremost, nurses might have felt pressured to participate against their wish. To avoid this, researchers stated clearly that participation was voluntarily and all findings would be treated anonymously. Secondly, to promote their own agenda, recruiters might have nominated nurses that shared their position regarding the research topic. However, we consider this unlikely, since the views expressed during focus groups were varied and reflected a broad range of opinions. Likewise, there is a risk of bias due to the perceived power imbalance between participants and an intensive care physician as moderator. This was sought to be minimized by moderating discussions neutrally, empathically, and respectful. A debriefing took place after each focus group between the two moderators, were these issues were addressed. Focus groups are useful to encourage participants to express their own views and identify norms and cultural values [13]. However, the mix of participants from two different wards could actually counteract this purpose by repressing minority views. To minimize this, group sizes were kept fairly small and moderators encouraged all participants to contribute with their views and experiences. Finally, abductive inference holds the risk of implying non-existing correlations between disparate phenomena. It is a limitation of the present study that the issue was not addressed by methodological triangulation to corroborate results (e.g. supplementing interviews with observations or surveys).

Despite these limitations the results of the study are valuable in identifying barriers and facilitators related to the EWS protocol at our institution in regard to crucial aspects of patient care. Namely: to identify at-risk patients through proper monitoring; escalation of care through collaboration between doctors and nurses; and definite treatment of severely ill patients by the MET.

## Conclusion

We identified a number of barriers and facilitators related to the use of the EWS system in regard to patient monitoring, collaboration with junior doctors, and MET. Adherence to monitoring frequency was considered an important part of good nursing practice, but would frequently be set aside during busy periods for other tasks. Collaboration and communication with doctors and MET about patients with elevated EWSwas generally not conducted according to protocol due to the high number of patients with these scores and negative feelings towards MET.

Generally, EWS performs well to predict outcomes in cohort of patients, but is less well suited to form the sole basis of clinical decision for individual patients. Also, it reduces the complex and dynamic interactions between individual co-morbidities, underlying disease processes, and response to treatment to a single number, with the inherent risk to overlook critical observations and subtle changes in patient conditions. Overall, it is important to remember that the system functions as a safety net, and is intended to complement, rather than substitute experience and good judgment. Its role is to alert staff to deteriorating patients and initiate individualized treatment; this is a collaborative task that requires both technical and non-technical skills to function smoothly. Implementation and maintenance of RRS, including EWS protocols and MET, should address these issues to optimize care for these patients.
